# A Non‐Metaproteomics Researchers’ View on Metaproteomics in Microbiome Research

**DOI:** 10.1002/pmic.202500019

**Published:** 2025-04-27

**Authors:** Velma T. E. Aho, Laure‐Alix Clerbaux, Anne Kupczok, Bree Tillett, Neha Garg, Jannie G. E. Henderickx

**Affiliations:** ^1^ Faculty of Biosciences Norwegian University of Life Sciences Ås Norway; ^2^ Laboratory of Hepato‐Gastroenterology, Institute of Experimental and Clinical Research UCLouvain Brussels Belgium; ^3^ Bioinformatics Group Wageningen University & Research Wageningen the Netherlands; ^4^ Frazer Institute Faculty of Medicine The University of Queensland Australia Brisbane Australia; ^5^ School of Chemistry and Biochemistry, Center for Microbial Dynamics and Infection Georgia Institute of Technology Atlanta Georgia USA; ^6^ Center for Microbiome Analyses and Therapeutics, Leiden University Center of Infectious Diseases (LUCID) Leiden University Medical Center Leiden the Netherlands

**Keywords:** functional analysis, metagenomics, metaproteomics, microbiome, multiomics

## Abstract

Metaproteomics, an emerging field among the omic techniques, holds great promise for unraveling the function of microbiomes in host health and our environment. Metaproteomics can also be a valuable addition to multiomics studies of the microbiome, complementing genome‐resolved metagenomics, metatranscriptomics, and metabolomics. The potential advancements from metaproteomics and multiomics research touch a breadth of disciplines, including ecology, biochemistry, immunology, medical microbiology, cell physiology, and medicine, and could lead to both fundamental and applied discoveries. However, there are significant roadblocks to widespread adoption of metaproteomics among microbiome researchers. In this Viewpoint article, we highlight the pivotal role of metaproteomics in microbiome research by showcasing its advantages, exploring opportunities to overcome challenges, and paving the way for its broader adoption as a mainstream technique. We hope that the recommendations provided in this Viewpoint article will inspire new, beneficial collaborations between proteomics experts, algorithm and infrastructure developers, biochemists, cell biologists, and microbiologists, enabling the construction of a knowledge base of microbiome function that can have an immediate and direct impact on host health and the environment.

## 1 Introduction

In recent years, the exploration of microbiomes and their functional implications has gained significant attraction in many fields such as ecology, human health, agriculture, and bioeconomy [1]. To this end, microbiome studies have predominantly focused on metagenomic next‐generation sequencing (NGS) techniques. Metagenomic shotgun sequencing is currently the most widely used method and provides information on the functional potential of a microbiome in addition to the information on phylogenetic distance and taxonomic composition. However, the taxonomic profiles are often yet to be associated with specific functional outcomes, which requires a comprehensive understanding of the proteins effectively translated. Moreover, species composition alone might not be always informative since the same functions can be encoded by different bacterial species [2]. Besides metagenomics, there is an array of omic techniques with which the function of the microbiome can be queried at various levels. For example, metatranscriptomics offers an additional layer of insight by enabling the study of the complete set of RNA transcripts expressed within a microbial community at a given time. Metaproteomics identifies and quantifies protein levels by mass spectrometry. Hence, metaproteomics provides direct insights into translated proteins within a microbial community, reflecting real‐time biological processes and enzymatic activities beyond what genomics or transcriptomics data can reveal. Metabolomics focuses on microbial metabolites and differs from metaproteomics by providing insights into the end products of metabolic pathways rather than the proteins that catalyze those processes. With the development of an array of omic techniques, standardization of microbiome studies is required to increase the comparability and integration of different types of microbiome studies.

During an interdisciplinary Lorentz Center workshop in Leiden (the Netherlands) in March 2024, which focused on “*Deciphering microbiome functions*,” metaproteomics was highlighted as a promising tool. At the same time, challenges in its application were acknowledged in a survey conducted amongst participating scientists who had less experience using metaproteomics. Leveraging omics technologies might bridge microbial identity and functionality, enabling the annotation of proteins of unknown functions and establishing more profound insights into microbial ecology. We believe that this is essential for advancing our knowledge in various domains, including personalized medicine, environmental sustainability, and biotechnology.

Here, we aim to report the advantages of metaproteomics in uncovering microbiome functions, while highlighting the challenges we encounter as non‐experts in implementing this approach within our genome‐resolved microbiome research. We conclude with recommendations for using metaproteomics to improve our understanding of the functional capabilities of microbial communities.

### 1.1 Metaproteomics: A Key Player in the Multiomic Toolkit

Metaproteomics can be a valuable addition to multiomics studies of the microbiome, complementing genome‐resolved metagenomics, metatranscriptomics, and metabolomics. First, metaproteomics can corroborate the functional potential of genomes based on sequence information by showing that the corresponding proteins are indeed synthesized [3]. This is useful as the genome‐based functional repertoire of the microbiome might not be representative of the synthesized proteins, which is dependent on, for example, resources available or the presence of other community members. Metaproteomics can reveal the variation of protein abundances across time and under different conditions, providing detailed insights into the functional dynamics and ecological niche of an organism [4]. Thus, even when bacterial communities are stable in their taxonomic composition or in their genome‐based functional repertoire due to functional redundancy, metaproteomics can yield useful insights into the functional activity. While metabolomics also uncovers microbial community dynamics and metabolic processes, the advantage of metaproteomics over metabolomics is that metaproteomics data can be resolved taxonomically, which is more challenging for metabolomics. Moreover, metaproteomics can also improve gene and functional annotation by detecting open reading frames that were missed by de novo protein prediction [5].

Metaproteomics also has several advantages compared to transcriptomic measurements, since it measures the protein abundances in situ where post‐transcriptional regulation is considered [6]. Additionally, proteins are more stable in the cell than RNA. This is particularly important for validating and improving metabolic reconstructions derived from genomic data and confirming if the synthesized proteins align with these predictions [7, 8]. Furthermore, metaproteomics can provide insights into co‐regulation and help to understand the regulation of changes in metabolic profiles observed via metabolomics, delineating co‐dysregulated pathways.

Finally, metaproteomics of host‐associated communities can measure host and microbial protein abundances directly without the need for adjusting protocols for transcriptomics that might rely on polyA enrichment (for the host) and rRNA depletion (for metatranscriptomics). For host‐microbe interactions, it can be crucial to measure the synthesized proteins directly (compared to transcript expression) to understand the bidirectional interactions between a host and its microbiome, the impact of microbial molecules on host receptors and metabolism, and the ways in which the host can modify and control its microbiome. In the context of the human gut microbiome, metaproteomics might help to assess the impact of the host on the changes in the microbiome functions and vice versa, for example, for enhanced hepatic lipid metabolism, impaired intestinal permeability, altered energy metabolism, or disrupted bile acid metabolism. Research that incorporates both host and microbiome layers has been described as “holo‐omics” [9, 10] and metaorganism research [11]. The use of metaproteomics can be highly beneficial in this expanding field of research [12].

### 1.2 Roadblocks for Widespread Application of Metaproteomics

The insight into biological processes and function provided by metaproteomic analysis is appealing for many researchers, particularly for translational research. However, there are significant roadblocks to widespread uptake among scientists. Although the roadblocks below are discussed in light of metaproteomics, not all apply to metaproteomics per se but rather reflect general problems related to omic techniques.

One roadblock is that metaproteomics is perceived to be more expensive than other functional omics. Recent advancements, such as multiplexing and short‐gradient methods, have reduced per‐sample costs [13], although instrument costs remain high. Unlike metagenomics and metatranscriptomics, there is a lack of commercial metaproteomic sequencing facilities providing metaproteomic analyses, so that research laboratories which lack expertise are often deterred from engaging in these types of experiments.

Moreover, metaproteomic sample preparation and analysis pipelines are often complex and overwhelming. Sample preparation is technically complex since the availability of commercial kits is limited. This often leads to batch effects in larger studies, as well as biases toward either Gram‐positive or Gram‐negative bacteria, depending on bacterial lysis protocols. Sample contamination from the environment (e.g., fatty acids, metabolites) is another challenge, as microbiome research has shifted from analyzing bacterial proteins in isolation to complex communities. Although this problem is not unique to metaproteomics, peptide acquisition is highly susceptible to sample degradation and to blockages from a build‐up of contamination over time, which can be more common in these complex environments. Automation and adaptations of standard approaches to high throughput, such as filter‐aided sample preparation (FASP), can help overcome these challenges [14].

In addition, lack of reproducibility in data‐dependent acquisition (DDA) can discourage non‐proteomic scientists from investigating these approaches. In traditional sample acquisition (DDA), fragment ion selection is used to identify peptides [15]. The stochastic nature of fragment ion selection, in which the most abundant ion is selected, biases protein identification. This also creates large amounts of missing data between samples. Determining whether proteins are truly absent from a sample or missing at random can be difficult. Corrective data adjustments will differ depending on the source of these missing values. Exactly which proteins are identified/missing will differ between runs. While samples can be fractionated with increasing depth and sequencing resolution, or run with technical replicates to increase reproducibility, this has the caveat of increasing analysis costs. New data‐independent sample acquisition (DIA) approaches reduce fragment ion selection bias by sequencing all ions in a defined range, known as a “dynamic range.” However, this approach produces chimeric spectra, which can challenge peptide assembly into proteins [16, 17, 18, 19]. Some strategies, such as the use of neural network algorithms by the tool DIA‐NN [20], successfully address this challenge. Recently, the metaproteomics initiative worked on a “Microbiologist's Guide to Metaproteomics” [21, 22] to provide non‐metaproteomics researchers an introduction to the field and strategies on how to handle missing data.

Arguably, the greatest challenge limiting the widespread application of metaproteomics is protein inference. Unlike metagenomic analyses, where nucleotide assembly is defined by highly constrained bonds, peptides can originate from many different proteins with alignment predicted by complex, often proprietary, algorithms, masking exactly how protein lists are assembled. As a result, exactly which proteins are called can differ between assembly algorithms and different commercial alignment software have license‐specific formats. An in‐depth explanation of this challenge can be found at Schallert et al. [23].

Protein identification is further challenged by the common requirement to search large publicly available databases (>10 million protein sequences), which can lead to false discovery limitations with many proteins remaining unidentified. As expected, there are many millions of bacterial species included in these generic databases. Constructing a study‐specific protein database from study genomic data can reduce the false discovery rate (FDR). However, deep metagenomic shotgun sequencing and the additional sample preparation increase cost; the latter being required as there are not yet established parallel extraction methods, although these are currently being developed.

Many research groups, which lack the means or the expertise to undertake these studies themselves, are increasingly using publicly available datasets. However, poorly recorded metadata can limit subsequent mining of protein sequences. There are currently initiatives underway to standardize metaproteomic metadata records [24, 25].

### 1.3 Future Directions and Opportunities

Despite the challenges, we believe metaproteomics offers many advantages and can be a valuable addition to multiomics studies of the microbiome, complementing genome‐resolved metagenomics. The steps that could be taken to pave the way for more widespread application of metaproteomics also provide many opportunities for collaboration across disciplines.

First of all, there is a need for better standardization of methods related to metaproteomics. This holds true for every step of the workflow, starting from optimizing sample storage conditions [26] and extraction protocols [27], all the way to harmonization of data formats [28] and establishing best practices for the repositories where data is archived [29]. Such standardization and harmonization would also be crucial for enhancing the comparability between studies. Furthermore, sharing laboratory and computational workflows through publications, online platforms (such as Zenodo (https://zenodo.org), Figshare (https://figshare.com), the Open Science Framework (https://osf.io), Galaxy [30] (https://galaxyproject.org/), WorkflowHub (https://workflowhub.eu/), or Protocols.io (https://www.protocols.io) and code repositories (e.g., Git‐based repositories for scripts and software) improves transparency by fulfilling the FAIR principles [31]. Such resources also offer a good starting point for researchers new to the field. Finally, community efforts to benchmark workflows, such as CAMPI [27] (https://metaproteomics.org/campi/), are an asset for the entire field, taking steps toward establishing methodological gold standards.

Another area where improvement is needed is in the incorporation of metaproteomics in multiomic workflows. Steps toward adopting metaproteomics in multiomic workflows by non‐experts could include improving methods for parallel extraction of different biomolecules (DNA, RNA, and protein) from the same sample, and robust, open source and easy to use software tools for integrating multiomic data. Collaboration between metaproteomic researchers and other experts in microbial omics is also crucial for establishing the best possible reference databases for metaproteomics. This is important for environments that are less studied, particularly if the option of using a study‐specific metagenome‐based database is not available due to cost restrictions [32]. Finally, another topic for collaborative research could be performing extensive and systematic comparisons between metatranscriptomic and metaproteomic datasets to better understand the relationships between mRNA and protein abundances in complex environmental samples.

Metaproteomics is still a young and developing discipline. To make sure that its promising methodology is used in the best possible ways, collaborations should also involve specialists representing the many fields that can benefit from metaproteomic data, ranging from medical and environmental science to biotechnological research and development. Current community efforts in metaproteomics (such as forthcoming CAMPI studies in multiomics, quantitation, and proteins of unknown functions) are already working to address many of the challenges outlined in this article and offer opportunities for the non‐metaproteomics scientists to become more involved in the field.

## Conflicts of Interest

The authors have declared no conflict of interest.

## Data Availability

The authors have nothing to report.
